# An objective index of walkability for research and planning in the Sydney Metropolitan Region of New South Wales, Australia: an ecological study

**DOI:** 10.1186/1476-072X-12-61

**Published:** 2013-12-24

**Authors:** Darren J Mayne, Geoffrey G Morgan, Alan Willmore, Nectarios Rose, Bin Jalaludin, Hilary Bambrick, Adrian Bauman

**Affiliations:** 1 Sydney School of Public Health, The University of Sydney, Sydney, NSW 2006, Australia; 2 Public Health Unit, Illawarra Shoalhaven Local Health District, Wollongong, NSW 2522, Australia; 3 Illawarra Health and Medical Research Institute, University of Wollongong, Wollongong, NSW 2522, Australia; 4 University Centre for Rural Health - North Coast, Sydney School of Public Health, The University of Sydney, Sydney, NSW 2006, Australia; 5 North Coast Public Health Unit, Lismore, NSW 2480, Australia; 6 Bureau of Transport Statistics, Transport for NSW, Haymarket, NSW 1240, Australia; 7 New South Wales Ministry of Health, North Sydney, NSW 2060, Australia; 8 Centre for Research, Evidence Management and Surveillance, Sydney and South Western Sydney Local Health Districts, Liverpool, BC NSW 1871, Australia; 9 School of Public Health and Community Medicine, University of New South Wales, Liverpool, BC NSW 1871, Australia; 10 Centre for Health Research, University of Western Sydney, Campbelltown, NSW 2560, Australia; 11 National Centre for Epidemiology and Population Health, The Australian National University, Canberra, ACT 0200, Australia

## Abstract

**Background:**

Walkability describes the capacity of the built environment to support walking for various purposes. This paper describes the construction and validation of two objective walkability indexes for Sydney, Australia.

**Methods:**

Walkability indexes using residential density, intersection density, land use mix, with and without retail floor area ratio were calculated for 5,858 Sydney Census Collection Districts in a geographical information system. Associations between variables were evaluated using Spearman’s rho (ρ). Internal consistency and factor structure of indexes were estimated with Cronbach’s alpha and principal components analysis; convergent and predictive validity were measured using weighted kappa (κ_w_) and by comparison with reported walking to work at the 2006 Australian Census using logistic regression. Spatial variation in walkability was assessed using choropleth maps and Moran’s I.

**Results:**

A three-attribute abridged Sydney Walkability Index comprising residential density, intersection density and land use mix was constructed for all Sydney as retail floor area was only available for 5.3% of Census Collection Districts. A four-attribute full index including retail floor area ratio was calculated for 263 Census Collection Districts in the Sydney Central Business District. Abridged and full walkability index scores for these 263 areas were strongly correlated (ρ=0.93) and there was good agreement between walkability quartiles (κ_w_=0.73). Internal consistency ranged from 0.60 to 0.71, and all index variables loaded highly on a single factor. The percentage of employed persons who walked to work increased with increasing walkability: 3.0% in low income-low walkability areas versus 7.9% in low income-high walkability areas; and 2.1% in high income-low walkability areas versus 11% in high income-high walkability areas. The adjusted odds of walking to work were 1.05 (0.96–1.15), 1.58 (1.45–1.71) and 3.02 (2.76–3.30) times higher in medium, high and very high compared to low walkability areas. Associations were similar for full and abridged indexes.

**Conclusions:**

The abridged Sydney Walkability Index has predictive validity for utilitarian walking, will inform urban planning in Sydney, and will be used as an objective measure of neighbourhood walkability in a large population cohort. Abridged walkability indexes may be useful in settings where retail floor area data are unavailable.

## Background

Walkability describes the capacity of built environments to support walking for multiple purposes [1] including utilitarian purposes such as walking for transport [2]. Active transport may contribute to environmental health, as well as to a population’s total daily physical activity [3-6]. Increasing local opportunities for transport-related walking through strategic land development and use is also a cornerstone of transport and urban policies, such as the Sydney Metropolitan Strategy [7]. This strategy focuses on the next two decades of urban development in Sydney, Australia, and identifies the need to design new urban growth to support active walking and cycling [7].

Walking for utilitarian purposes is associated with the built environment attributes of proximity of destinations, mixed land use, connectivity and population density [2,5,8-10]. Proximity and land use mix are inter-related planning and urban design constructs. Proximity describes the distance between different land uses, such as employment, retail and residential, and is defined by two variables: density and land use mix [5]. Density refers to the concentration of land uses within physical space and land use mix describes variation in the patterning of co-located land uses. Neighbourhoods that are compact and have heterogeneous land use encourage walking by reducing the distance between origins and destinations [1,5], while higher population densities provide the critical mass to support a range of destinations within a small area [2]. Connectivity describes the directness of walking routes between origins and destinations using street and pedestrian networks and infrastructure, and has a direct effect on proximity [5]. Connectivity is maximised by traditional grid-based networks as they provide more direct and greater choice of routes resulting in more proximal residential and non-residential destinations [2].

Objective measurement of the built environment is increasingly undertaken within geographical information systems (GIS) using spatial data [2] to derive composite measures that characterise the walking typology of geographic areas [1,11,12]. These composite walkability indexes are used to capture the natural co-variation between built environment variables, address multicollinearity issues in statistical models, and facilitate communication of results [2]. They also have a number of benefits over perceived walkability self-report measures. Objective measures have smaller measurement errors, can be compared across studies and are easier to translate into health and planning policy [13,14]. Indexes derived using GIS may also be retrospectively applied to historical data.

Two frequently utilised GIS indexes are the South Australian Physical Activity in Localities and Community Environments (PLACE) study [1] and North American Neighbourhood Quality of Life Study (NQLS) [12] walkability indexes. These indexes use GIS to operationalise four built environment variables: net residential density; street connectivity; land-use mix; and net retail area (a measure of pedestrian friendliness). The raw scores for each variable are standardised using either deciles [1] or Z scores [12], which are summed to give a total score for each spatial unit and then divided into quartiles corresponding to low (quartile 1) through high (quartile 4) walkability. Both the PLACE and NQLS indexes have high specificity for utilitarian walking; correlate with health outcomes and behaviours; have demonstrated construct validity; can be calculated for areas; and are the basis for a growing body of walkability research in Australia and internationally [12,15-20]. The use of these four-attribute indexes is often limited though by the availability of retail floor space data, which is difficult to source [1,12] and frequently unavailable [21] for index construction. Applications of abridged indexes that exclude retail floor area ratio may allow greater use of walkability indexes in research [22-25]; however, research on the comparability of associations between three and four-attribute indexes and domain-relevant outcomes is required, especially if evidence is to be synthesised across studies using full and abridged indexes.

The strategic and research aims of developing a Sydney Walkability Index (SWI) were to influence urban planning through the Sydney Metropolitan Strategy [7]; using the Sydney Walkability Index will enable planners to assess and measure the walkability of existing and developing built infrastructure. In addition, the Sydney Walkability Index was developed concurrently with the baseline recruitment of a large population-based cohort of older adults, the 45 and Up Study, comprising 267,000 persons aged 45 years and over and living in New South Wales (NSW), Australia [26]. Two thirds of this cohort are resident in Sydney, and future work by our group will compare the walkability index described in this paper with self-report environmental attributes, derived from the PANES questionnaire [27], and examined in relation to weight change, physical activity change and morbidity and mortality measures collected in the 45 and Up Study and its three year follow up (SEEF study) [27,28].

The primary research aims of this paper are to: compare two forms of a Sydney Walkability Index with three and four environmental attributes; examine the validity of a three-attribute Sydney Walkability Index as a measure of walkability when retail floor space data for a four-attribute index are not available; and examine the relationship of the Sydney Walkability Index to regional rates of active travel assessed through reported walking to work in the 2006 national Census. A secondary aim of the paper is to describe the spatial patterning of walkability across the Sydney Metropolitan Region using the Sydney Walkability Index.

## Methods

### Study area

The Sydney Walkability Index was based on the Sydney Metropolitan Region of Australia, which covers an area of 3685 km^2^ and had a population 3.7 million in 2006 [29]. Walkability indexes were also calculated for the Sydney central business district (City of Sydney local government area), which had 156,521 residents in 2006 and a land area of 26.7 km^2^[29].

### Index construction

The Sydney Walkability Index was based on the PLACE index [1], which was selected because it forms the basis of a growing body of walkability research. Index values were calculated for 2006 Australian Census Collection Districts and temporally referenced to calendar year 2007 to coincide with the midpoint of the baseline data collection of the NSW 45 and Up Study [26]. Census Collection Districts are the smallest statistical output areas used to report demographic data from the 2006 Australian Census of Population and Housing, and aggregate up to larger administrative units such as postcodes and local government areas [30]. There were 5,858 inhabited Census Collection Districts in the Sydney Metropolitan Region in 2006, with a median land area of 0.2 km^2^, 200 residential dwellings and 550 residents.

Walkability was initially operationalised as a composite of four environmental attributes:

a. Residential dwelling density—the number of residential dwellings per square kilometre of residential land use

b. Intersection density—the number of intersections with three or more road junctures per square kilometre of total land area

c. Land use mix—the entropy of five land use classes (residential, commercial, industrial, recreational and other uses) divided by the ratio of each Census Collection District’s land area to the smallest (1,752 m^2^) in the study region to adjust for differences in the size of spatial units [17]

d. Retail floor area ratio—the amount of retail floor area in square metres divided by the total amount of commercial land use in square metres

Residential dwelling density, street network connectivity and land use mix characterise urban design, density and diversity, while retail floor area ratio is indicative of pedestrian-orientated design [12]. These attributes have been consistently associated with walking behaviour in the research literature, especially for utilitarian purposes [2,18].

Environmental attribute variables were calculated using geographic and spatial information systems for each Census Collection District using digital boundaries from the Australian Bureau of Statistics [30]. Data describing residential dwelling locations were obtained from a local utility provider; land use from the New South Wales Department of Planning and Infrastructure; road centrelines from the New South Wales Department of Land and Property Information; and retail floor area from the Property Council of Australia and City of Sydney council. The distribution of each environmental variable was divided into deciles, scored from 1 (lowest) to 10 (highest), and the scores summed to give a total walkability index score. The Sydney Walkability Index was then split into quartiles to reflect low, medium, high or very high walkability. Associations between area-level characteristics, environmental variables and Sydney Walkability Index scores were assessed using Spearman’s rank-order correlation coefficient (ρ) as variable distributions were highly skewed.

The fourth attribute, retail floor space, was only available for the central business district [31]. We therefore calculated two walkability indexes: a full four-attribute walkability index only for the City of Sydney comprising residential dwelling density, intersection density, land use mix, and retail floor area ratio; and an abridged three-attribute index for both City of Sydney and the entire Sydney Metropolitan Region that excluded retail floor area ratio.

### Index validity and reliability

The convergent validity of the abridged index to the full index was assessed using the 263 City of Sydney Census Collection Districts. The square of Spearman’s rank order correlation coefficient (ρ) was used to calculate the proportion of variance in the full index score that was retained by the abridged index, and whether this was higher than the 75% expected *a priori* given the abridged index used three of the four variables of the full index. Weighted kappa (κ_w_) was used to assess agreement between walkability quartiles assigned to Census Collection Districts by the abridged and full indexes.

Cronbach’s alpha was used to assess the internal consistency of the full and abridged Sydney Walkability Indexes. Principal components analysis was used to evaluate the latent variable structure of indexes calculated for the City of Sydney and Sydney Metropolitan Region areas. Analysis was performed using the Spearman correlation matrix of environmental variables for each index. Eigenvalues greater than 1 were used to select the number of retained components and pattern values greater than 0.3 were used to identify items loading on extracted components.

The predictive validity of the full and abridged indexes for utilitarian walking was evaluated using data on the number of people reporting walking entirely to work (i.e. using active transport) at the 2006 Australian Census [29]. Data for employed adults 16 years and over within each Census Collection District that walked entirely to work on the 2006 Census day were summarised by abridged walkability index score decile and also by abridged walkability quartiles stratified by median household income to control for the inverse association between walkability and socioeconomic status [32] and for consistency with previous index validation studies [12]. Logistic regression was also used to assess the independent effect of walkability on the likelihood of walking to work above that attributable to age, sex, socioeconomic status and population density [15,18,19]. The odds of walking to work in medium, high and very high walkability areas were estimated relative to low walkability areas after adjusting for area-level median household income, percentage working population male, percentage working population aged 16–24, 25–34, 45–54, 55–64 and ≥65 years, and population density per square kilometre. This analysis was undertaken for the entire Sydney Metropolitan Region using the abridged index, and for City of Sydney using both full and abridged indexes. Only the prevalence of walking entirely to work could be estimated because mixed mode trips involving walking are not reported in the Census.

### Walkability patterning

Choropleth maps were used descriptively to display geographic variation in the distribution of walkability and component environmental variables for the entire Sydney region using the abridged walkability index. Evidence of clustering in walkability maps was assessed using Moran’s I, a global measure of spatial autocorrelation that indicates the extent to which areas with similar attribute values are co-located in space [33]. A Moran’s I of 0 indicates the absence of spatial patterning, while values greater than 0 indicate clustering of areas with similar attribute scores and values less than 0 indicate clustering of areas with dissimilar attribute scores.

Non-spatial statistical analyses were performed using SAS 9.2 software, an alpha level of 0.05 and two-tailed significance tests. Geo-processing, mapping and spatial statistical analysis were undertaken in FME 2010 SP4 and ArcGIS Desktop 9.3.1 software packages.

## Results

### Index construction

Environmental data provided sufficient coverage and resolution for the calculation of the three-attribute abridged Sydney Walkability Index for all 5,858 inhabited Census Collection Districts in the Sydney Metropolitan Region. A retail floor area ratio indicator and full walkability index were also calculated for the 263 of 311 (84.6%) inhabited City of Sydney Census Collection Districts.

### Item correlations, internal consistency and principal components

The upper and lower diagonals of Table 1 show correlations between population density, built environment indicators, and walkability indexes for Census Collection Districts in City of Sydney and Sydney Metropolitan Region areas, respectively. Medium to large correlations (range: 0.41 to 0.76) were observed between population density and all environmental indicators except retail floor area ratio, which were unrelated. Medium to large associations were also observed between land use mix, residential dwelling density and retail floor area ratio (range: 0.33 to 0.66). All environmental variables were highly correlated with full and abridged walkability index scores but were strongest for residential dwelling density and land use mix in City of Sydney and for residential dwelling and intersection density in Sydney Metropolitan Region. Large correlations with full walkability index scores were observed for all built environment indicators (range: 0.58 to 0.89) but were on average 10% higher for Sydney Metropolitan Region compared to City of Sydney local government area except for land use mix, which was 13% lower.

**Table 1:** Spearman’s rho correlations between population density, environmental variables and walkability indexes

	**Population density**	**Residential dwelling density**	**Intersection density**	**Land use mix**	**Retail floor area ratio**	**Full index score**	**Abridged index score**
Population density	1.00	0.76^†^	0.41^†^	0.42^†^	0.00	0.58^†^	0.70^†^
Residential dwelling density	0.82^†^	1.00	0.23^†^	0.51^†^	0.16^*^	0.70^†^	0.78^†^
Intersection density	0.77^†^	0.66^†^	1.00	0.26^†^	0.14^*^	0.60^†^	0.66^†^
Land use mix	0.24^†^	0.44^†^	0.26^†^	1.00	0.33^†^	0.78^†^	0.79^†^
Retail floor area ratio	--	--	--	--	1.00	0.59^†^	0.28^†^
Full index score	--	--	--	--	--	1.00	0.93^†^
Abridged index score	0.76^†^	0.89^†^	0.80^†^	0.69^†^	--	--	1.00

Upper diagonal shows data for the 263 City of Sydney Census Collection Districts and lower diagonal shows data for the 5,585 Sydney Metropolitan Region Census Collection Districts.

^*^p < 0.05, ^†^p < 0.0001.

Internal consistency as assessed by Cronbach’s alpha was 0.60 for both the full and abridged City of Sydney indexes and 0.71 for the abridged Sydney Walkability Index. Principal components analysis extracted a single component for each walkability index, which explained 46.3, 62.4 and 64.2 per cent of the variability in City of Sydney full, City of Sydney abridged and Sydney Walkability Index environmental variables, respectively. Table 2 shows the pattern loadings for environmental variables on each index component.

**Table 2:** Pattern loadings for full and abridged walkability indexes

	**Full index**	**Abridged index**	
	**City of Sydney**	**City of Sydney**	**Sydney walkability index**
	**(n = 263)**	**(n = 263)**	**(n = 5858)**
Residential dwelling density	0.75	0.81	0.90
Intersection density	0.56	0.59	0.82
Land use mix	0.82	0.82	0.66
Retail floor area ratio	0.55	--	--

### Convergent validity

The abridged and full walkability index scores for City of Sydney Census Collection Districts were highly correlated. The abridged index explained 87% of the variability in the full index score, significantly more than the 75% expected *a priori* (p < 0.0001, see Table 1). There was also good agreement between the walkability classifications assigned to each district by the two indexes, especially for low and high quartiles. The weighted kappa coefficient for their cross classification was 0.74 (95% CI 0.69–0.79), and all districts were assigned a walkability quartile by the abridged index within one category of that assigned by the full index.

### Predictive validity

**Figure 1:**
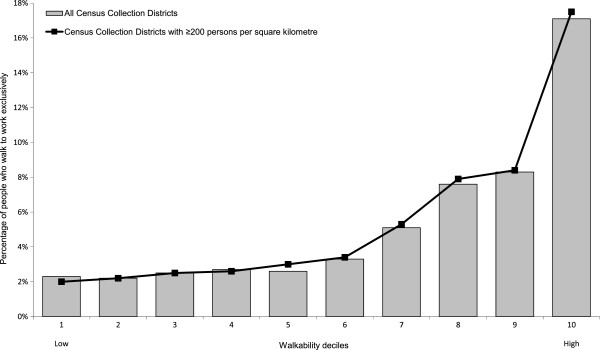
Prevalence of walking to work in Sydney Metropolitan Region by Sydney Walkability Index score decile.

The grey bars in Figure 1 show the relationship between decile of abridged walkability score and prevalence of reporting walking to work at the 2006 Australian Census for the entire Sydney Metropolitan Region. The percentage of employed persons who walked to work increased with increasing area-level walkability. The magnitude of the increase was small until the sixth decile, after which increases in prevalence became more pronounced for each successive increase in area-level walkability. We initially considered that this threshold effect may be due to the inclusion of a high number of relatively low density spatial units in the index construction. However, an almost identical profile was obtained when index construction was limited to Census Collection Districts with population densities ≥200 persons per square kilometre as suggested by Leslie et al. (represented by the line series in Figure 1) [1].

**Figure 2:**
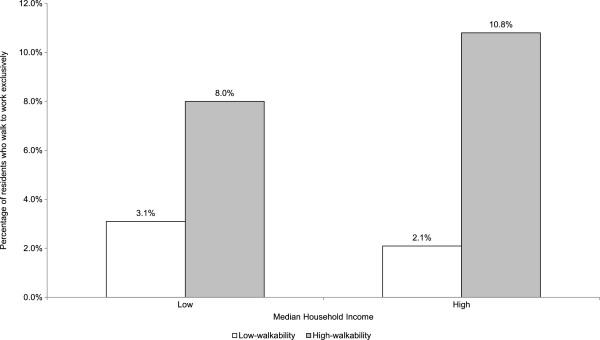
Prevalence of walking to work by walkability and median household income in Sydney Metropolitan Region.

Figure 2 shows the prevalence of walking entirely to work in the Sydney Metropolitan Region for the lowest and highest abridged walkability quartiles stratified by area-level median household income. For both low income and high income strata the percentage of people who walked to work is higher in high walkability areas compared to low walkability areas, although the prevalence ratio (PR) was twice as high in high income (PR = 5.2) areas compared to low income areas (PR = 2.6). Prevalence of walking to work in high income-high walkability areas was 3.1 percentage points higher than in low income-high walkability areas but just under one percentage point (0.9%) higher in low income-low walkability areas compared to high income-low walkability areas.

Odds ratios for walking to work for the entire Sydney Metropolitan Region by abridged walkability quartiles are reported in Table 3. The unadjusted odds of walking to work increased significantly with increasing walkability $\left(\chi_{3}^{2}=3241.37,p<0.0001\right)$ and were 5.75 times higher in high walkability areas compared to low walkability areas. Adjusting for demographic and socioeconomic covariates attenuated odds ratios; however, the odds of walking to work were still three times higher for high compared to low walkability areas, and the strong exposure-response relationship between walkability and prevalence of walking to work remained highly statistically significant $\left(\chi_{3}^{2}=861.47,p<0.0001\right)$. Table 4 shows the results of this analysis replicated for the 263 City of Sydney Census Collection Districts for which both full and abridged walkability indexes were available to assess any additional explanatory power of the full index. Adjusted parameter estimates for this comparative analysis were very similar, with full index effect sizes just 1–10% higher than abridged index associations and comparable exposure-response relationships.

**Table 3:** Associations between area-level walkability and prevalence of walking to work in Sydney Metropolitan Region (n = 5,585)

	**Frequencies**			**Unadjusted**		Adjusted*	
	**Walked to work**	**Employed**	**Percent**	**Odds ratio**	**95% confidence interval**	**Odds ratio**	**95% confidence interval**
Walking category							
Low	10068	434391	2.3	1.00		1.00	
Medium	9143	350333	2.6	1.13	1.03–1.24	1.05	0.96–1.15
High	17486	378057	4.6	2.04	1.88–2.22	1.58	1.45–1.71
Very high	37224	310277	12.0	5.75	5.33–6.20	3.02	2.76–3.30

*Adjusted for population density and area-level median household income, percentage working population male, and percentage working population aged 16–24, 25–34, 45–54, 55–64 and ≥65 years.

**Table 4:** Comparison of adjusted associations between prevalence of walking to work and area-level walkability for full and abridged indexes (n = 263)

	**Full walkability index**		**Abridged walkability index**		**Difference in odds ratios (%)**
	Adjusted* odds ratio	**95% confidence interval**	Adjusted* odds ratio	**95% confidence interval**	
Walking category					
Low	1.00		1.00		
Medium	1.57	1.24–1.98	1.43	1.13–1.81	9.9
High	2.11	1.65–2.68	2.01	1.59–2.55	4.6
Very high	2.64	2.07–3.38	2.62	2.02–3.40	0.8

*Adjusted for population density and area-level median household income, percentage working population male, and percentage working population aged 16–24, 25–34, 45–54, 55–64 and ≥65 years.

### Walkability patterning

**Figure 3:**
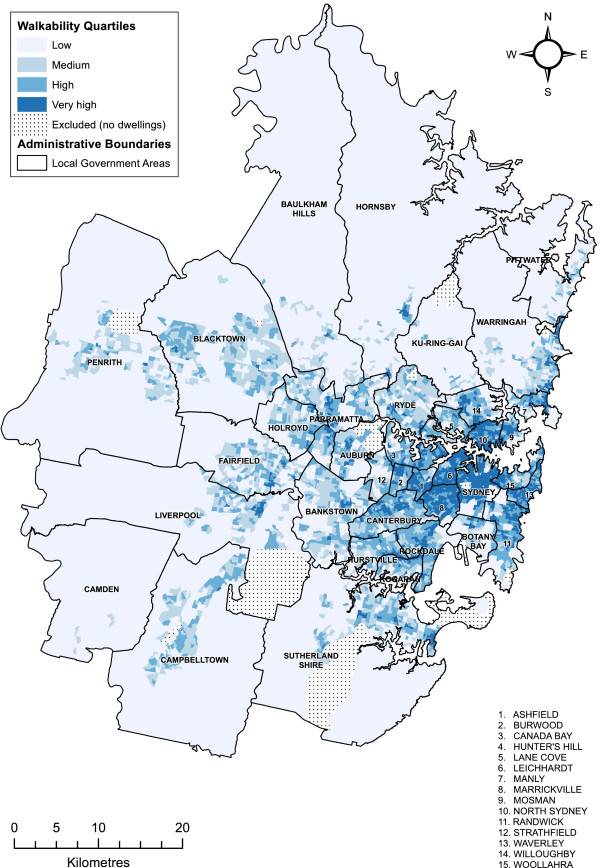
Distribution of Sydney Walkability Index quartiles in Sydney Metropolitan Region.

The geographic distribution of abridged Sydney Walkability Index quartiles for the Sydney Metropolitan Region is shown in Figure 3. Abridged index scores were strongly associated with residential density and displayed a clear east–west gradient (see Table 1), as did index component environmental variable scores (not shown). High walkability was most concentrated in eastern and central Sydney with progressively lower levels in western and outer suburbs where the population is sparser. Stippled areas indicate the 32 uninhabited, non-residential Census Collection Districts excluded from calculations. Moran’s I for the map in Figure 1 was 0.73 (Z = 93.47, p < 0.0001), which indicates walkability is highly clustered with areas of similar walkability more likely to be proximal than distal.

## Discussion

This study validated a walkability index for Sydney that was comparable to the PLACE index frequently used for walkability research [1]. The PLACE index combines four built environment attributes associated with walking for utilitarian purposes: residential dwelling density, intersection density, land use mix, and retail floor area ratio. A limitation of this and similar four-attribute indexes is that floor space data are frequently unavailable to calculate retail floor area ratio [21]. This was the case in the current study for which floor space data were only available for a part of the study region. We therefore tested a three-attribute abridged index and found it to have similar measurement properties to a full index. This has international implications because retail floor area data are often difficult to source [1,12] or unavailable [21] for index construction and applications of abridged indexes that exclude retail floor area ratio may allow for greater use of walkability indexes in research [22-25].

The innovative observation in this study was that the abridged walkability index retained 87% of the variability in the full index, assigned all analysis units to within one walkability quartile of the full index, and found associations of similar magnitude to the full index between walkability and prevalence of walking to work after adjusting for demographic and socioeconomic confounders. Thus, in the absence of retail floor space data, an abridged index comprising residential dwelling density, intersection density and land use mix only may be used to characterise walkability. This would be advantageous in the many global locations where retail floor space data are not available [21]. We recommend researchers with data on the four walkability components in only a subset of spatial units also compare three and four-attribute indexes to further validate this finding.

Principal component analysis of the abridged Sydney Walkability Index attributes extracted a single component with high loadings for all attributes; similar component structures and loadings were also observed for City of Sydney full and abridged indexes. This appears to be the first time that a latent variable structure of a PLACE/NQLS index has been described, and supports the validity of the Sydney Walkability Index as a cohesive measure of walkability. Internal consistency of the abridged Sydney Walkability Index is also acceptable for research purposes [34], especially given the small number of items included in the index [35].

These results demonstrate the feasibility of a Sydney Walkability Index, the utility of a three-attribute derived index, and a consistent relationship between walkability and walking to work that is only partially moderated by socioeconomic status. Walking to work increased monotonically with increasing abridged walkability index score decile, and was higher for high walkability areas compared to low walkability areas in both lower and higher income areas. These findings concur with NQLS index validation outcomes that found increasing walk trips with increasing decile of walkability, and more walking in high versus low walkability areas for both high and low income strata [12], providing additional support for the validity of the abridged Sydney Walkability Index.

Although the prevalence of walking to work in the Sydney Metropolitan Region increased with increasing walkability decile, this association was more pronounced at the upper deciles of walkability. Excluding low population density Census Collection Districts as suggested by Leslie et al. [1] did not alter this trend, and may indicate homogeneity in the distribution of urban sprawl outside the inner city area. This is consistent with the adjusted odds for walking to work, which were significantly higher for high and very high walkability areas compared to low walkability areas, but similar for medium compared to low walkability areas. Further study into possible walkability threshold effects may provide useful information for planning and policy interventions to improve built environments to support walking.

Visualisation of choropleth maps indicated consistent patterns of clustering across the study area for Sydney Walkability Index scores and its component environmental variables. This was supported by correlation analyses that indicated all variables were strongly associated with one another. High residential density, street connectivity and land use mix were concentrated in the central, eastern and north Sydney areas, and decreased along an east–west gradient to a ring of low walkability areas on the outer fringes of the Sydney Metropolitan Region. This patterning is consistent with the spatial distribution of population density and socioeconomic disadvantage in the study area [36], and highlights the planning potential of the Sydney Walkability Index to target walkability infrastructure upgrade and development initiatives in the Sydney Metropolitan Region.

Understanding the features of the built environment that facilitate or constrain walking is important for research, planning and policy aimed at increasing the proportion of adults who attain recommended levels of physical activity [5]. Linking the Sydney Walkability Index to land use and transport planning strategies such as the Sydney Metropolitan strategy [7] has the potential to create more walkable communities, and have a greater population impact on reducing physical inactivity than individual-level interventions [5,37].

Spatially referenced objective walkability measures such as the one constructed here may also be linked to existing administrative or epidemiological data collections with location information to add both research and policy value. For example, the Sydney Walkability Index is being used in the 45 and Up study to profile the independent health effects of environmental factors such as walkability, to compare with self-report (PANES) items, and to assess changes in activity behaviours when mid to older aged adults change residence [26,38]. From the Sydney urban planning perspective, objective indexes of the built environment could also be used to monitor, inform and evaluate policy through desktop simulations of proposed developments for walkability based on their urban design features, identify “best buy” areas for infrastructure upgrades and residential development to maximise active transport use, and monitor changes in the walkability of geographical areas over time and following environmental interventions [1,39]. In this regard, the Sydney Walkability Index provides an “out-of-the-box” resource for researchers, planners and policy makers that is evidenced-based and derived using the best-available spatial data.

### Limitations

The main limitation of this study is that comparability analyses between full and abridged walkability indexes were confined to the City of Sydney local government area as it was the only area for which retail floor space data were available. It is feasible that the similarity in performance of three and four-attribute indexes is unique to this area and may not be as comparable in other areas. However, the generalizability of our results beyond the City of Sydney area is supported by our corresponding analysis for the entire Sydney Metropolitan Region, which produced similar associations between walkability and prevalence of walking to work using the three-attribute index, and identified similar factor structures and explained variance for the Sydney Metropolitan Region abridged index. It would be advantageous for researchers to confirm this finding in other cities where data are available for all four walkability components.

Another limitation of this study is that GIS derived estimates of walkability were not compared to the physical reality on the ground via site visits, so the level and nature of any measurement error is unknown. Previous studies using similar indexes have included field verification as the indexes were used to generate sampling frames for interventions [1,12]. Field validation in these cases comprised “informal windshield observations” [12] and systematic observations [1]. While both studies observed some discrepancies in walkability classifications, Leslie et al. concluded that the PLACE index had good face validity and that field observations were concordant with index classifications for the majority of their study units [1].

## Conclusions

The abridged Sydney Walkability Index is comparable to existing indexes that include retail floor area ratio and has demonstrated predictive validity for utilitarian walking. Greater use of validated indexes for environment-behaviour research will improve study comparability and inform urban planning and policy to improve the walkability of communities.

## Competing interests

The authors declare that they have no competing interests.

## Authors’ contributions

DJM undertook GIS and statistical analyses and wrote the first draft of the manuscript with contributions from GM and AB. AW processed restricted-access residential dwelling and land use spatial data to produce anonymised aggregate data used in environmental variable calculations. All authors contributed to and have approved the final manuscript.
